# Miscellanies

**Published:** 1839-01-01

**Authors:** 


### MISCELLANIES.

---

### Researches on Suppuration. By George Gulliver, Esq. Assistant-Surgeon to the Royal Regiment of Horse Guards.

The object of this paper is to shew the *Frequent Presence and the Effects of Pus in the Blood, in Diseases attended by Inflammation and Suppuration.* The paper was read before the Royal Society on the 14th of June, 1838, and is contained in the Philosophical Magazine for September last.

As Mr. Gulliver observes, the humoral pathology has latterly begun to reassume some consequence, and, although it is not likely ever to appear in its ancient form, or to be invested with its former importance, still there can be little doubt that it has been undeservedly neglected.

The facts which have principally contributed to direct the attention of physiologists to the state of the blood, have been the cases of purulent deposits after injuries and operations, or in connexion with phlebitis. They have gone far towards establishing the probability of a vitiated condition of that fluid, as a leading morbid state, and have rendered the existence of pus in it a more than plausible conjecture. We look on the paper before us, which establishes the fact, as a highly important one, and as forming the commencement of a series of enquiries, which will, perhaps, exert a great influence in medicine.

Mr. Gulliver has detected pus in the blood on numerous occasions and under various circumstances. He details the steps of the examination, which was very simple, partly chemical and partly microscopic.

"Those who are acquainted with the minute constitution of the animal fluids are aware of the rapid and energetic action of water on the blood-corpuscles: now the globules of pus undergo no change after having been long kept in water; accordingly, if the suspected blood be mixed with this fluid, the blood-corpuscles will soon become invisible, and any globules of pus that may be present will subside to the bottom of the vessel, and may be easily seen, and their characters determined, with a good microscope. Ammonia instantly renders the blood-corpuscle invisible, while that of pus is acted on but slowly by the alkali; and the different action of acetic acid on pus and blood is equally remarkable. Hence I have employed these agents advantageously in conjunction with the other means; and I have also seen pus-globules in the blood, though rarely, without any preparation. With water, however, the examination is most easy, simple, and satisfactory, if the observer be thoroughly familiar with the microscopic characters of the fluids under examination. A good instrument, nevertheless, is necessary; and the admirable deep object glass of Mr. Ross is the one I have principally employed. It is hardly necessary to add, that chyle-globules are not likely to be mistaken for those of pus, since, independently of other distinctions, the medium diameter of the latter is at least $\frac{1}{2000}$ths of an inch, which is about twice that of the former."

Mr. Gulliver details, in a very brief manner, seven experiments and eleven cases. We shall quote all the experiments.

*Exp.* 1.—A weak solution of corrosive sublimate was injected into the subcutaneous cellular tissue of a dog's thigh; great swelling of the limb took place, and he died forty-five hours after the injury. A good deal of serum mixed with fibrine was found in the cellular tissue of the thigh, but there was no purulent deposit.

Several pus-globules were detected in some blood obtained from the right ventricle of this dog's heart.

*Exp.* 2.—A large dog had both his tibiæ injured by some operations connected with necrosis; great swelling of the limbs, with violent fever, succeeded, and he died forty-three hours subsequently.

A large quantity of fibrine was found effused into the cellular tissue of the extremities, mixed, in one of them, with a very scanty proportion of purulent matter.

In some blood, obtained from the vena cava, numerous globules of pus were observed.

*Exp.* 3.—An irritating fluid was injected into the peritoneum of a dog; he had great thirst, refused food, and died the third day after the operation.

A large quantity of coagulated lymph and sanguinolent serum with some pus was found in the belly.

In some blood obtained from the inferior cava vein many globules of pus were seen.

*Exp.* 4.—Two ounces of pus were injected into the left pleura of a dog, and very carefully confined there; he was thirsty and feverish for fifty-five hours after the operation, when he was killed.

An ounce of fluid, almost entirely serum, was found in the pleura, and some fibrinous exudation on the membrane.

Blood from the heart, as well as from the vena cava was examined, and found to contain several pus globules.

*Exp.* 5.—Four ounces and five drams of pus were injected into the peritoneum of a dog, and the wound carefully closed; he died thirty-seven hours after the injury.

There were only nine drams of a sero-sanguinolent fluid found in the peritoneum, and a considerable quantity of coagulated lymph on the membrane.

Pus was detected in the blood.

*Exp.* 6.—Half a dram of pus, mixed with half an ounce of water, was gradually injected into the crural vein of a dog.

Some fever followed, and he refused solid food for two days, but recovered at the end of a week.

The same quantity of pus was soon afterwards injected into the other crural vein, when similar symptoms were produced, and he perfectly recovered in a few days.

*Exp.* 7.—Six drams of pus having been injected into the crural vein of another dog, he was not much affected at first, but in a few hours became very weak, was stupid, thirsty, and refused his food. After thirty hours he took but little notice of surrounding objects, his respiration was hurried, and he died thirty-six hours after the operation. In the blood of the inferior cava some pus-globules were readily detected.

We pass to the cases.

*Case* 1.—A girl died of confluent small-pox on the ninth day of the disease. There was great swelling of the integuments.

In the blood of the right ventricle numerous pus-globules were found.

*Case* 2.—A woman had confluent small-pox, uncomplicated with erysipelas or inflammation of the viscera.

On the eighth day of the disease some blood was drawn from a vein in the arm: several pus-globules were found in this blood.

*Case* 3.—A male child, æt. 15 months, died on the ninth day of small-pox. Only a few pustules appeared, and these were imperfectly developed: there was considerable swelling in the face, slighter in other parts.

At the post-mortem examination, it was observed that a small quantity of a white opaque fluid might be squeezed from the cut surfaces of the lymphatic glands of the neck and groin: this fluid had the microscopic and chemical characters of pus.

In some blood obtained from the right ventricle and from the inferior cava vein, pus was detected.

*Case* 4.—In a woman who died of puerperal peritonitis, the peritoneum contained a large quantity of coagulated lymph, serum, and purulent matter.

Pus was detected in the blood obtained from the right ventricle of the heart.

*Case* 5.—James Green, æt. 27, was admitted into hospital with an ulcer of the leg. Seven days afterwards, the limb began to swell, and there was hardness in the femoral vein, with some redness in the course of the absorbents on the inner side of the thigh. The swelling of the limb increased gradually; he had first pain in the head, thirst, and quick pulse; then purging, pain in one wrist, with restlessness, incoherency of speech, and offensive breath; finally, low muttering delirium, accelerated respiration, and coma preceded his death, which took place on the 12th day after his admisssion into hospital.

At the post-mortem examination, the large veins of the limb were found to be occluded throughout by firm clots of blood, mixed with pus and coagulated lymph, and the lining membrane of the femoral vein was in many places of a red colour, and coated with fibrine. In the iliac vein no such signs of inflammation appeared, although there was a large coagulum of blood, which had lost its red color, containing in its centre a small quantity of matter resembling pus. Several purulent deposits presented in the sheath of the femoral vessels, and in the intermuscular cellular substance.

The matter resembling pus in the clot of the iliac vein had neither the chemical nor microscopical characters of that fluid.

In some blood obtained for examination from the right ventricle and from the vena cava, numerous globules of pus were found.

*Case* 6.—J. H. æt. 22, had a superficial wound of the tibia, succeeded by much swelling of the limb, and effusion of fibrine and sanguinolent serum. A few pus-globules were found in blood from the cava.

*Case* 7.—M. J. æt. 42, had erysipelas of the face, succeeded by jaundice, and effusion of an ounce of turbid serum, with a little purulent matter into the right pleura, and eight ounces of sanguinolent serum into the left. Some blood was obtained for examination from the larger veins, and found to be greatly contaminated with pus.

*Case* 8.—Sergeant Dunn, æt. 29, had profuse suppuration between the muscles and beneath the integuments of the thigh; he died, after some weeks' suffering, exhausted by hectic.

The purulent matter was extremely offensive, putrefying with great rapidity, and sometimes coagulating spontaneously, when set aside for a short time. It was poor in true pus-globules, but contained a large quantity of flaky fibrinous matter, to which its opacity was chiefly owing. Many pus-globules were found in the blood, obtained from the right ventricle.

*Case* 9 was one of pulmonary phthisis. In the blood obtained from the vena cava and right ventricle, many pus-globules were found.

*Case* 10 was one of irritative fever from a large abscess behind the trochanter femoris. An ounce of blood was drawn by cupping from the neighbouring sound parts, and some pus was detected in this blood.

*Case* 11.—In a horse who died with vomicæ, and sero-purulent fluid in one pleura, pus was detected in the blood of the vena cava inferior.

Mr. Gulliver has found pus in the blood in other instances. Dr. Davy has also found pus in the blood in consumptive cases. The latter gentleman, indeed, has found pus in the blood in seventeen instances after death, in sixteen of which there was declared suppuration, and in one none could be detected: in the latter, the patient died of acute inflammatory disease.

Mr. Gulliver offers some observations on the formation of pus and on the nature of suppuration. But we believe that he intends to prosecute this subject. We shall not, therefore, touch on it at present, but content ourselves with laying before our readers, the conclusions which Mr. Gulliver draws from his experiments and cases.

"The term suppurative fever is not new, and its signification is probably now extended; for it seems to be an appropriate one for the different forms of constitutional disturbance under consideration. If the presence of pus in the blood and the fever in these cases be not related as cause and effect, the coincidence would appear to be no less interesting than remarkable.

What a field of inquiry this view opens to us! Henceforth, whenever a patient is affected with inflammatory fever, or that low typhoid state which is so generally a fore-runner of death, as a consequence of traumatic or idiopathic inflammation, the state of the blood will present an interesting subject of investigation. And this is not merely a matter of curiosity; for the question will arise, whether, in the treatment of such cases, it would not be advantageous to produce suppuration as soon as possible on the surface of the body, so as to establish a drain by which the blood might be deprived of the offending matter. It may be asked also, whether the benefit so often effected by blisters, setons, and issues, in certain internal inflammations,—or by incisions which cause suppuration, in inflammatory affections of the integuments, be not explicable by this theory? It is well known that in cases of traumatic or idiopathic inflammation, attended with great swelling and febrile excitement, the establishment of suppuration in the part is generally a favourable symptom, the separation of the pus from the blood being a sort of crisis to the symptomatic fever. In small-pox, it is a popular belief that 'the striking in,' as it is termed, or suppression of the pustules, is a bad symptom; and this is so far true, that the worst cases of this disease are those in which there is great swelling of the integuments without the due formation of pus in the usual situation. In every instance in which I have examined it, I found pus in the blood of patients affected with small-pox.

In the fourth and fifth experiments the pus which was injected into the serous sacs would appear to have been absorbed. A more careful inquiry, however, would be requisite to warrant this conclusion; for in some experiments made by Dr. Davy, the quantity of matter injected seemed to be increased; and I have since made an experiment with the same result.

The absorption of pus being the cause of hectic fever, is an old hypothesis, which the detection of pus in the blood in cases of chronic abscess and in pulmonary consumption might be supposed to confirm. It does not seem necessary, however, to assign two causes for one effect. When pus in large quantities is incessantly forming in the capillaries, it is easy to imagine how it may become mixed with the blood.

I have related instances of pus in the blood, independently of suppuration out of the vessels : this fact appears to be of some importance, for it must be inferred that the pus was not absorbed, but formed in the blood.

If it be objected to some of the foregoing views, that pus and extravasated blood are often absorbed without any ill effects, and that no constitutional disturbance may ensue after inflammation and the consequent effusion of fibrine—it may be remarked, first, that pus and blood are probably absorbed in a modified state; and secondly, that a small quantity of pus, like other poisons, gradually added to the circulation may not be productive of bad symptoms. The sixth and seventh experiments may be cited in illustration. It is probable that the degree and type of the fever induced by the presence of pus in the blood may be found to depend on the extent to which it may be contaminated."

He adds :—

"I cannot conclude this paper without expressing a hope that it will lead to a still more careful and extensive examination of the blood in various diseases than has hitherto been attempted. The microscope may become as important an instrument to the pathologist, and even to the medical practitioner, as the stethoscope. If my results should be confirmed, it is hardly too much to expect that some important discovery, particularly in diagnosis, may be made by a patient investigation of the blood in many malignant diseases, such as cancer : it is not long since the urinous fever, as it is called, was found to depend on the accumulation of urea in the blood."

We think it would be premature to offer an opinion on these facts. They call for further investigations, for we are probably only on the threshold of our knowledge of the morbid alterations of the fluids. Mr. Gulliver appears a very zealous young surgeon, and deserves all possible encouragement.

---

### On the present State of the Law of Lunacy. By a Barrister.

We wish that none of the laws of the land were in a worse condition than the Law of Lunacy! A great cry has lately been made against this law on account of some individual and solitary abuses—accidents to which all laws, however wisely framed, are liable. Let us see what the law is :—a person supposed to be of unsound mind is subjected to the separate examinations of two medical men. If then two men are of opinion that the individual is deranged, he can be sent to, and received into a licensed lunatic asylum, where he is, from time to time, visited by the commissioners of lunacy, whose duty it is to see that no man is confined an hour after he evinces symptoms of sanity. Oh, but say the croakers, the two medical men may be ignoramuses, and incapable of distinguishing between a sound and unsound mind! No doubt there are many ignorant men in our, as well as other professions. But allowing that the two who sign a lunatic certificate are ignoramuses, how many *lives* must be annually jeopardized by such practitioners, and yet we have no outcry against these, or even against arrant quacks! But they may be bribed to give false evidence. This is a very likely story. The detection of their ignorance or knavery would immediately follow, and then they would be liable to prosecutions and be ruined for ever! But the croakers have many strings to their bow. Although the commissioners may prove a salutary check on the confinement of sane persons by unprincipled friends and falsifying doctors, yet the temporary imprisonment, between committal and the visitations of commissioners, must drive sane men mad! Was there ever such a statement put forth by people who were actually sane themselves?

From long acquaintance with the subject, and mature reflection on it, we are convinced that, for one sane person who is illegally, of course, committed to a lunatic asylum, there are one hundred insane persons, from false delicacy, or amiable prejudices, permitted at large, till their property is injured or their own lives destroyed by their own hands. Every multiplication of the forms to prevent improper confinement will only multiply the number of suicides, increase the destruction of property, and extend the sphere of domestic misery. Those who fix their eyes on a few isolated cases of misdemeanor, on the parts of keepers of asyla, entirely overlook the far greater evil of deterring friends from, restraining the actions of insane persons, involving their families in ruin, and ultimately winding up the drama by suicide. The reflections that are thrown so lavishly on the testimony of medical men, in their certificates—and on the cruelty exercised in lunatic asylums towards the insane, are, for the most part, false, and the rest are grossly exaggerated. In the course of twenty years of extensive observation, we have never met with a single instance of either of the above delinquencies—in this country at least. Men, whether in or out of the profession, are apt to study their own interests. The physician seldom tries to keep his patients ill by way of increasing his fame and fortune—the grocer will not be ready to insult his customers, on whom he depends for support—nor will the tavern-keeper maltreat the guests who are dining or drinking under his roof. Neither will the medical man risk his reputation and livelihood by signing a false certificate of insanity, knowing, as he must, that the first visitation of the commissioners may blast his professional character for ever, and even subject himself to a prosecution. Nor will the keepers of lunatic asylums—who are themselves in a state of sanity—harass or irritate their inmates—or impose restrictions that are more than necessary for the safety of those committed to their charge. And this humanity is dictated by prudence—or, if you please, by SELF-INTEREST. For, assuredly, whoever act otherwise, (and some will do so, as in other departments of life) will very soon have empty-walls instead of well-filled apartments. In fine, the whole outcry is founded on morbid sensibility, false views of human nature, want of experience and observation—or love of notoriety and propensity to blunder.

---

### LAW OF LUNACY. THE MONTHLY CHRONICLE.

We have been gratified by the perusal of the leading article in the above able journal, for December—entitled the "TREATMENT OF INSANITY IN ENGLAND." The following extract will shew how completely the writer coincides with the sentiments which we have stated in a short article on this subject in the present number of the Journal.

"Granting for a moment that two medical men could be found who would thus, for a consideration, put their reputation in jeopardy, and destroy for ever their professional respectability, is it likely that, however little they might care about their character, they could be readily drawn into an act, the commission of which would subject them to be indicted for a misdemeanor? Our own impression is, that medical men are too well aware of the responsibility of their position to allow themselves to be led into any such dilemma, and still less to become participators wilfully in such a fraud; but, for the sake of argument, let it be assumed that the wicked relation has succeeded in procuring two abandoned instruments to assist him in his nefarious project. What follows? The supposed lunatic is carried to an asylum. Now, the proprietor of the asylum, or the regular medical attendant of the asylum, must also be drawn into the plot, or it fails just at the point when its completion is nearly effected. The ingenuity, therefore, and the corrupt influence of the chief mover of this complicated drama must be fairly irresistible if he can succeed in gaining over his new adherent, without whose aid all that he has previously effected goes for nothing. But here again—to give the utmost latitude to circumstances—let us suppose that the has gained his ends, that he has borne down the scruples of two professional men, and of the proprietor or medical attendant of an asylum, and induced them all to place themselves in a situation of serious danger, for the sole purpose of helping him to effect his iniquitous object; he has yet to overcome the greatest difficulty of all. After he has gained over the medical men to sign a false certificate, and then prevailed upon the proprietor or medical superintendent of an asylum to receive and detain the individual against their conviction of his sanity, he must get three commissioners in lunacy (not even having the power of selecting them, as the case must be decided in the order of visitation, of which he cannot, by any means in his power, acquire any previous knowledge) to sanction the unhallowed transaction!"

It is to be recollected, however, that the guards and fences against unlawful and unnecessary detention respect public and private "ASYLUMS" only. There is a great flaw—a great oversight in the Act, which permits the friends of a certified lunatic to be kept in private lodgings, without any surveillance of the commissioners, and without any return being made to the commission, till twelve months after the date of confinement! Here is the grand grievance in the law of lunacy! We cannot better describe this evil than in the words of our talented contemporary :—

"In the asylum there is a perpetual check upon the attendants; and there is constant variety of some sort to break the uniformity,—new faces, a succession of incidents marking the progress of time, and supplying topics to divert and carry off the speculations of the lunatic out of himself,—all of which are essential to his restoration. In the private lodging there are no resources of any kind, except the visits of a physician, brief, perhaps, and irregular: the same face, identified for ever with unchanging stupor, distraction, or coercion, is constantly presented to the unhappy invalid: he looks around for relief in vain; he languishes for something to give a fresh aspect to the scene; and, in this terrible want, cast in upon himself, he feeds upon his delusions, and grows wilder and more intractable day by day, or else sinks into utter imbecility. His keeper, left alone with this demented man, adopts, partly in fear, and partly for his own ease, a system of unnecessary restraint. To him it is an existence of continuous deprivation. He longs also to be at liberty, and may possibly snatch an interval of escape, every now and then, taking care in the meanwhile to make such provision for the safe custody of his charge as shall effectually prevent any accident from occurring. But the uninterrupted intercourse of a sane and an insane person, thus confined to a single room, is productive in the end of fearful consequences. The keeper, after exhausting whatever benevolence he may possess in fruitless attempts to reconcile the patient to his situation, becomes morose, jaded, and harsh—perhaps vindictive. His nature has not been practised in self-subjugation—an authority is entrusted to him over a superior—he has the whole management in his own hands—and how far he may abuse his trust depends upon his moods and his constitution. Sometimes it occurs that keepers so circumstanced gradually take the tone of the despairing solitude, and lose their power to meet the exigencies of their position; and instances have actually happened in which they were removed in consequence of visible evidences of approaching madness.

From this outline the choice between the two existing modes of providing for the insane may be determined. If the asylums, in the loose phraseology of the day, may be designated mad-houses, the isolated retreats of individuals may, with great propriety, be described as mad-lodgings."

We are free to admit, also, that, even in the licensed asylums there is room for improvement in the laws that regulate them. Thus it is to be feared that the visitations of the commissioners are not sufficiently frequent—and more especially as relates to the first visit after confinement—which should, we think be within seven days after the restraint on personal liberty. The qualifications, too, of the keepers of asylums are not sufficiently inquired into. It is needless to expect perfectibility—or rather perfection, in these or other functionaries; but, at present, there is no standard by which to test their knowledge of the disease, their experience in its treatment, their education, moral character, &c. &c. It would also be a judicious enactment, that the keeper or proprietor of an asylum should reside within its walls, or in immediate contiguity, instead of delegating the supervision to other and irresponsible agents. The number of patients, too, ought to be regulated, not by the dimensions of the mansion, but by the number and efficiency of the attendants. This limitation of patients would be productive of vast advantages, as conducing, among other things, to more accurate statistical details than have hitherto been obtained.

We recommend a perusal of the article alluded to, most strongly to our professional brethren.

---

### Hæmatophobia, or the Beauties of Continental Practice.

Our learned contemporary (the British and Foreign) has recently introduced us (in his peculiar laconic mode) to some German worthies, whose productions we shall probably take the trouble to examine next quarter, were it only from curiosity. These worthies are Messrs. Weitzlar, Schneider, and Simon. The works of the two latter are, as usual, only made known to us by name—but fortunately the titles alone of these tomes give us a tolerable insight into their nature. The one is called "*Hœmatomania,*"—the other "The Vampirism of the Nineteenth Century."

These titles speak for themselves. Dr. Weitzlar is less extravagant in his title-page, but apparently as unmeasured in his denunciations against the Lancet of England, and the Leech of France, as his hæmatophobic countrymen. "The new Italian school (says our contemporary) and the active treatment of England are his peculiar aversion." The death of Mad. Malibran, by homœopathy, too, is brought forward as an illustration and proof of hæmatomania in this country!! We find no fault with our "Cousin-Germans," or our confreres of France, in denouncing the malpractices of their own brethren; but when they come to stigmatize the therapeutics of this country, without being at all acquainted with the physical differences of constitution between them and us, resulting from diet, climate, manners, &c. they deserve to be castigated and exposed for their ignorance. Who would have dreamt of exhibiting calomel and black-strap to the Bayaderes in the Strand, when they were indisposed? Yet there is scarcely more difference between the food and the physique of a German and an Englishman, than between that of a Hindoo and a European. The Germans travel very little in England—the English know more of Germany than the Germans themselves. Who that has rambled from Trieste to the Baltic—or over almost any part of that long route, can have failed to observe the physiological, or rather pathological effects of sour-crout, apple-wein, sausages, tobacco, garlic, rancid oil, and a thousand unutterable, or, at least, undigestible things, that are daily crammed down the throats of our German neighbours? And what are those effects? They are readily cognizable by two of the senses—sight and smell. The rows of German teeth are like rows of stockades half-burned down, and black, in most places, and often every second stake entirely demolished—long before the age of 40 years! The breath is a very compounded exhalation, redolent of garlic from the stomach, tobacco from the lungs, and putrid effluvia from carious teeth. The complexion is tallowy, but well adapted for the growth of mustachios, and the stature, in a great majority of the middle and lower classes, is stunted. For the fidelity of this portrait, we appeal, not to our Anglo-Germanic literati, who seldom travel beyond the walls of their libraries, and who know about as much of Germany and the Germans, as they do of the dismal swamps in the centre of Australia; but to those who have actually visited the soil of our Saxon forefathers. Taking, then, the diet and constitutions of our continental neighbours into consideration, can we come to any other conclusion than that the same methodus medendi will not answer, at least in extent, for them and for the beef-eating Britons? The difference of constitution on the North and South sides of the English Channel is proved by diseases as well as by remedies. See the havoc produced by cholera in Vienna, Naples, Berlin, and Paris, compared with London and the great towns in England. Even in Scotland and Ireland, where the diet is not so good as in England, the cholera was more severe. We do not, therefore, quarrel with, or criticise, the practice of the Germans, in their own country, but we consider it a piece of great impertinence, complicated with ignorance, that they should set themselves up as censors of British therapeutics, seeing that they are either unable or unwilling to estimate the difference of climate, constitution, diet, &c. existing in the two cases.

If these observations be true, and we think it would be difficult to disprove them, we may appreciate the inutility, nay, the mischief, of attempting to import the *practice* of the Continent into this country, and *vice versa*. How our British brethren have fared in their practice amongst the Germans and French, we will not pretend to say. We suspect that small is the number of Napoleons and rix dollars which they have brought back to these islands. But this we are enabled to say, from pretty extensive observation—that not one in five of those who have studied or sojourned much on the Continent, have ever succeeded in this country afterwards. Of this fact we have seen such numerous and melancholy examples, that we have no doubt about it. We throw out the hint to those whom it may concern. To ourselves it is of no importance; but we warn the rising aspirants to transcendental physiology and pathology to take care how they imbue their minds with the timid and *hæmatophobic* therapeutics of our German brethren.

---

### Saratoga Springs.

These waters have obtained a prodigious reputation in North America, and are resorted to by myriads of valetudinarians of all descriptions. We see that they are about to be imitated at Brighton, by that indefatigable chemist and mineral water manufacturer, Mr. Schweitzer.

The ingredients are potassa, soda, ammonia, lime, magnesia, strontia, proloxide of iron, ditto of manganese, alumina, silica, carbonic acid, nitric acid, sulphuric acid, iodine, bromine, chlorine. This is certainly a splendid bill of fare, but it must be remembered that the dishes are of the most homœopathic dimensions. Thus, in 1,000 grains of the water, there are only about five grains and a half of the aggregate medicinal agents—scarcely enough to give the waters a taste or flavour of physic. And of these 5½ grains, 3 are composed of soda and chlorine—nearly one of carbonic acid—leaving about 1½ grains for the whole of the other constituents. We have no doubt that all true disciples of Hahnemann will flock to this spring at Brighton, and direct their patients to take, each morning, a drop of this elixir diluted in six beakers of the natural element.

---

MEDICAL REFORM; BEING THE SUBJECT OF THE FIRST ANNUAL ORATION, INSTITUTED BY THE BRITISH MEDICAL ASSOCIATION, AND DELIVERED AT THE SECOND ANNIVERSARY OF THAT SOCIETY. By A. B. GRANVILLE, M.D. 8vo. Price One Shilling. Sherwood and Co. Nov. 1838.

This is one of the best things which Dr. Granville has published—though written, *currente calemo,* the orator having only a few days to prepare his subject. It is a masterly analysis of medical abuses, drawn from the evidence delivered before the parliamentary committee. The materials are lucidly arranged under three distinct heads, viz.—1. *What is there to reform?* 2. *How far has reform hitherto progressed?* 3. *What yet remains to be done for the accomplishment of a total reform?* These three questions he answers well, and supports them by answers of numerous witnesses called before the parliamentary committee. The pamphlet is so important in matter, and so cheap in price, that it ought to be disseminated through every ramification of medical society. Nevertheless we shall give an interesting extract from the third question, in which is embodied the plan of reform recommended by the author. Our readers will see that all its main points have been long advocated in the pages of this Journal.

"*What yet remains to be done to accomplish a total Reform?*

On this concluding part of my oration, time will only allow me the utterance of a very few words. But, though brief, I shall endeavour to be explicit, that we may be neither misunderstood nor misrepresented by the enemies of medical reform.

To accomplish this great, this all-important act, England, as one of the great national families of Europe, has only to place itself on an equality with the most enlightened among those nations. At present she stands alone, in the chaotic condition of her medical institutions. In no other part of Europe is the life of a fellow-creature, when invaded by disease, committed to the charge of three differently educated and differently qualified medical practitioners. Let him be poor, or let him be wealthy—lowly of condition, or sitting on high—the victim of disease, in all parts of the Continent, is sure to have by his bedside an attendant to whom an uniform system of education has imparted the utmost knowledge in his profession which a wise government could provide for him. And as for any distinction of rank among such attendants, the laws having prescribed the same education for, and granted the same qualifications to all—leave it to public opinion to establish it. In order to obtain readily results like these, the continental governments have provided one central medical faculty for the whole kingdom; with one or two branches where the territory is too vast, as in France for example. That faculty is directed to apply the same and the maximum test of examination to all who desire to practise the healing art. It is, moreover, invested with the power to recognise all persons who have proved themselves able to practise, and to grant to all such the same privileges, to be enjoyed by them in every part of the kingdom, unmolested by any secondary or delegated power. That such a measure of equality and protection on the part of the regent faculty may be justified, the nature and length of education, preliminary as well as medical, of *all* the candidates to be examined in the healing art, have been defined by special laws, which are not made subject to perpetual and capricious variations on the part of subordinate authorities. On the other hand, education itself is made accessible to the most moderate fortunes; and the final examination or inquiry into the proficiency of the students and candidates for degrees takes place in open courts, and not in a private conclave. The examiners do not elect and perpetuate themselves in secret; neither are they remunerated by the fees of candidates. Hence two sources of abuse or corruption are avoided. There may be corruption where there is secrecy, self-perpetuation, and irresponsibility on the part of the examiners. There may be corruption when, by secret proceedings, large sums of money are obtained from the many for distribution among the few. But, according to the continental system, such species of corruption cannot obtain.

After these preliminary observations, I proceed to offer to the members of the British Medical Association the opinion of their fellow-member who has had the honour of addressing them on the present important occasion, regarding the best mode of effecting their wishes; but I do not call upon them for a pledge of their adhesion to it. That opinion goes to declare that the great Act of medical reform in England will never be thoroughly accomplished, until the following great points shall have been conceded to the profession, and their execution secured by parliamentary statutes.

1st. A maximum degree of education, theoretical as well as practical, both preliminary and professional, obtained either at the existing colleges, or through authorised private teachers, for all medical students.

2ndly. The same uniform, and the highest possible test of qualification, for all who intend to practise the healing art, no matter in what branch; the said test to consist of practical as well as theoretical demonstrations of the candidates' abilities, exhibited at one or more public examinations, to be carried on in writing as well as verbally.

3rdly. One and the same rank and title in the profession bestowed on all who have proved themselves capable to exercise the healing art by the highest possible test of qualification: whether the candidate chose (choose) afterwards to practise as physician, or as surgeon, or both, or as one and the other comprising obstetricy, and any other subdivision of the art and science of medicine:—according as his own taste or inclination, or the strength of circumstances, and the situation he may be placed in, or the opinion of the public, may induce him to act:—thus affording to the poor, and the moderately affluent, as well as to the rich, (the lives of all of whom are of equal value in the eyes of humanity and the laws) the same means, and those of the highest character, for resisting the fatal inroads of disease.

4thly. An equal enjoyment of all the privileges and benefits appertaining to the highest degree of education and qualification as certified in a diploma, by every one possessing such a testimonial, in whichever part of Her Majesty's dominions he may choose to settle as a practitioner.

5thly. The establishment of One Faculty in the capital of each of the three realms—to be governed by the same laws—to be similarly constituted—and to be endowed with similar powers of qualifying candidates to practise in every part of the empire. As each of the capitals has its university for instructing and examining and granting degrees to students in every branch of educational knowledge, their privileges and rights should be left undisturbed in every respect, except as to the right of examining and conferring degrees in the medical art,—which must be surrendered to the medical faculty.

6thly. The medical faculty in each capital should consist of a certain number of eminent practitioners and public teachers, no matter to what particular branch of the profession they may have deemed it convenient or useful to confine themselves. By this provision, candidates would be certain to be examined in all the branches of medical art and science, by persons known to be thoroughly conversant with those branches. The members should be remunerated by a fixed salary, and not by fees dependent on the number of examinations; and to the post of member of the faculty all medical practitioners should be deemed eligible, either by open election or by competition.

7thly. The medical government should be centralised in the three faculties, so as to form but One Body, acting together in the framing and promulgation of the laws which are to regulate the profession—in defending the rights and interests of the latter—in superintending the medical police of the country—and in protecting the public from the ignorant and the pretender. The faculties should also have the power to establish, with the concurrence of the respective committees of governors, new and uniform regulations for the management of hospitals and the attendance of the medical officers and students, as well as for the appointment of the former, which should in future be open to competition on a public trial of skill.

8thly. The establishment of a board is likewise absolutely necessary, to consist of members of the faculty most conversant in chemistry, botany, and natural history, for the purpose of examining and licensing the venders of drugs, and compounders or dispensers of medicines. The same board should be empowered to fix, and from time to time to alter, the regulations by which the operations of the vending chemists and druggists ought to be governed.

9thly. A general registry of all who have been admitted to practise the healing art, as well as to sell and compound drugs, should be strictly kept at the faculty's offices, open to public inspection: so that in case of impostors or unqualified persons, (whose names of course would not appear in the said registry,) being found engaged in practising medicine in any of its branches, or in administering or compounding medicines, or in vending drugs, whether simple or compounded, with any reference whatever to health or disease—a *common informer* may be able to prove the fact by a mere reference to the registry, and convict the transgressor before a magistrate, who shall be empowered and bound to treat the case summarily, and by such pecuniary or other punishment as is awarded in cases of misdemeanor.

10thly. A law should also be enacted by parliament to prevent the sale of poisonous substances, and of all potent medicines by the licensed chemists and druggists, except on the prescription of a well-known medical practitioner.

11th and lastly. Those parts of the acts or charters under which the present medical corporated bodies or colleges claim the right to examine candidates, before the latter can be authorised to practise either physic, surgery, or pharmacy; and all such other acts in existence as interfere with the carrying out of the principles of legislation laid down in the present scheme of medical reform,—should be annulled. But in no other respect should the said medical corporate bodies be disturbed, nor any of their vested rights encroached upon. Their interference with the medical education, qualifications, degrees, and right to practise, of individuals, being once put an end to, the colleges should be permitted to continue the career for which they were originally intended,—that of promoting medical science, through and with the assistance of their halls, their libraries, and their museums. And inasmuch as the said colleges, whether in London, Edinburgh, or Dublin, or elsewhere, were founded for public and not for private benefit; and some of them are even now, or have been, supported by grants of public money; their respective establishments for the promotion of science should be thrown open to the public.

The best and wisest measure which the three corporate bodies in London can adopt under such circumstances, is to form themselves into a Royal Academy of Medicine, divided into three great classes, of medicine, surgery, and pure pharmacy. No doubt but that their example would be followed by the chartered medical bodies of the other capitals and cities. Each class should be limited in its numbers, and have a simple form of government; and all the members of the profession should be deemed eligible to a place in the academy, by election. This academy might become the medical consulting Board of the Government in matters of medical science."

### THE LATE MR. LOCKLEY.

For eighteen months or two years before his death, Mr. Lockley was in the habit of occasionally consulting Dr. Johnson respecting an affection of the heart, which was evidently of an organic nature, being an enlargement of structure and irregularity of function. Dr. J. warned Mr. L. repeatedly against excitement of mind, corporeal exertion and full diet, seeing from the habit of body and the configuration of the frame altogether, that apoplexy, as is often the case, would be likely to result from the cardiac disease. Mr. L. however, was very fond of the pleasures of the table, and it is well known that an eminent physician, whose name we shall not mention, advised him to drink a pint of claret daily, and to live generously. This advice being far more congenial to Mr. Lockley's disposition and taste than the frigid counsel of Dr. J. was, we have good reason to believe, adopted. In respect to the fatal termination at last, we have no doubt that the fall in Euston-square was the first effect of pressure on the brain, and that the pressure augmenting on the road, the sickness, insensibility, stertorous breathing, and other phenomena of apoplexy followed regularly in train. It was at this period, of course, that depletion offered any chance of success—and the more so, as the *cause* of the apoplexy was in the heart, and not, in all probability, an old standing disease of the brain, its vessels, or its coverings.

In respect to the melancholy circumstances which attended Mr. L's death, had they merely related to the feelings of *friendship* between two medical men —or the coldness or warmness of heart which might have predominated in the transaction, we should not have considered it our duty to advert to them in a public journal, or sit in judgment on the moral conduct, much less the conscience of one of our brethren. But unfortunately the very nature of the tragedy has called forth an intensity of excitement, and a freedom of comment, that have never before been equalled, on the death of a medical man.

The physician's conduct, on this occasion, extends widely beyond the circle of a private transaction between patient and attendant—or between host and guest. It becomes public property, as it involves impoitant ethical principles, relating to society in general, and to the profession in particular, as we shall presently shew. The consideration of this tragical event may be divided into three heads —1st. As is regards the principal or surviving personage,—secondly, as it relates to the medical profession itself—and thirdly, as it relates to a particular class of the profession.

1st. Sir Henry Halford has stated that he stands acquitted, *in foro conscientiæ*, of any neglect of duty towards his patient—of attention to his friend—or of moral obligation to humanity. We are glad of this, on his own account; for, assuredly the "still small voice of conscience" can derive little consolation from the voice of the public, which has, without a single exception, conveyed severe censure on his conduct.

We dwell on this head of the subject with great reluctance; but fortunately the portrait which we have drawn of the President of the College, in our number for July last (p. 194-5) puts it out of the power of our bitterest enemy to impute to us the shadow of personal, political or professional pique towards that eminent physician. The event, however, affords one more proof of retributive justice, even on this side of the grave. For many years past, the learned head of our College has annually mortified, or rather *abased* (by implication at least) his less fortunate and less aristocratic brethren, by extolling the "high tone of moral feeling," instilled into the minds of collegiate "FELLOWS," at Oxford and Cambridge! And what has been the result of this sermonizing for years before bishops, priests, ministers of state, and the elite of the aristocracy:—a very general burst of censure on the "tone of moral feeling," evinced on the late melancholy scene at Tring! This may prove a warning to those who, like the Pharisee of old, lift up their eyes to Heaven, and thank God "that they are not like other men."

We confess that we admire, rather than envy the moral feelings and the elastic conscience of the patron, the physician, and the host, who could abandon the friend, the patient, and the guest, at an obscure village—when stricken with apoplexy, and incapable of helping himself! We suspect that the unfortunate President (for we think him infinitely more unfortunate than Mr. Lockley) has found it easier to reconcile his own conscience, than the conscience of the public, to the species of "high moral feeling," displayed on the journey to Leicestershire. It was well known that the deceased was in bad health—was the father of two families—was in anything but affluence—and that his children depended almost entirely on his daily professional avocations. These considerations alone would have induced many an ignoble and obscure member of our profession, who had not had the advantage of a University education, to sacrifice a few hours, for the sake of administering professional assistance to an apoplectic brother practitioner. We shall add but one word more to this head of the subject—an appeal to Sir Henry's charity—if not his conscience—in behalf of the large and unsettled family of the deceased. The Baronet's ample means, his extensive influence, and his powerful eloquence, may yet gladden the widowed heart, sustain the helpless orphan,—and make the sod press lighter on poor Lockley's grave!

2ndly.—The MEDICAL PROFESSION has experienced a wound in the late transaction on the rail-road. Hitherto it has been almost universally acknowledged, at the bar, in the pulpit, and in public speeches, that medical men were distinguished by their liberality, as regarded professional attendance on their brethren, and the families of their brethren, independently of their exertions in behalf of the poor. Although exceptions should not affect general rules, yet there is no doubt but our enemies will be always ready to bring forward this memorable event, and thus by aiming a blow at the HEAD, inflict a wound on the BODY of the profession. The following question has been already asked—and until it be answered in the *affirmative*, the stigma on the profession will remain. "If a prince of the blood—a peer of the realm—or a bishop from the bench, had been in Lockley's situation, would the President of the College and the representative of the medical profession have left him at Tring in a fit of apoplexy, to be carried two miles in an omnibus, and then to have the sage advice of an apothecary's apprentice?" There is but one person in existence who can answer this question in the affirmative—and we are very much inclined to think HE could not do so!

3rdly.—No evil, we believe, ever occurs without some attendant good, sooner or later. Were it otherwise, "chaos would come again." The late tragical end of Mr. Lockley will open the eyes of the public, and of parliament to the "wisdom of our forefathers"—the ridiculous and mischievous system of medical education which trains one man for physic, another for surgery, and a third for pharmacy—each comparatively ignorant of the others' departments, though there is no natural boundaries between them, and it is perfectly evident that *all* the departments should be *equally* taught and learnt, by every member of the profession, however he may choose to practise afterwards. In other words,—every medical man should be ABLE to practise physic, surgery, and pharmacy—though he may not be *willing* to do so when he leaves the schools. But what is the case now? A valuable member of society falls down in a fit—there is a cry for a doctor—and lo, there is one on the spot. Every one presses to know what ought to be done. The doctor says the patient must be bled. Instantly the clothes are stripped off—the arm bared—and some female unties the ribbond from her waist to compress the veins of the patient. But the doctor exclaims, "not so fast good people. I can tell how much blood should be taken—but I can't bleed. And even if I could bleed, I dared not do so, because it is against the laws of my college." This is a precious state of things! But whenever the subject of medical education comes before parliament, the transaction at Tring may and will form an excellent illustration of the incongruous, absurd, and injurious laws which now regulate the instruction given to medical students.

### Dialogue between a Physician and a Physiologist; or Mechanism *versus* Vitalism.

*(Scene a Laboratory.)*

*Physician.*—Is it true, my dear Magendie, as reported in your lectures, that you can produce *inflammation* in the dead body?

*Magendie.*—I can.

*Physician.*—The cardinal points or phenomena of inflammation, as laid down by all writers, from Celsus to the present time, are rubor, tumor, calor, dolor.

*Magendie.*—Very well.

*Physician.*—Can you produce *rubor* in the dead tissues, and if so, by what means?

*Magendie.*—Nothing more easy. I dip the parts in red ink, or inject the *vessels*, with red wax.

*Physician.*—The tumor?

*Magendie.*—By injecting the *cellular* membrane with warm fluids of any kind.

*Physician.*—The calor?

*Magendie.*—I immerse the parts in hot water, or hang them before the fire.

*Physician.*—Humph! These are very mechanical processes for producing the first three phenomena of inflammation!

*Magendie.*—Doubtless. But all Nature's processes, in health or disease, are mechanical, quite as much so as those by which I imitate her operations.

*Physician.*—There is one phenomenon more, however, which I think will pose you. How do you produce the dolor?

*Magendie.*—"There you are in the clouds and fogs of vitalism—clouds and fogs by which you strive to conceal your ignorance. Sir, I admit the existence of no phenomenon or process in the living or in the dead body, but what can be made cognizable to the senses. We have no other inlets of knowledge than through the five senses. Now Sir, I ask you, can you *see* pain? No. Can you *touch* pain? No. Can you *hear* pain? No. Can you *smell* pain? No. Can you *taste* pain? No. Then, Sir, what monstrous absurdity is it to talk of a phenomenon presenting itself, you say, in a patient before you, and of which you cannot learn the most minute iota by the evidence of your own senses! Sir, I repudiate, scorn—nay, detest, all those phenomena and explanations which are founded on vitalism,—and thus I throw them to the winds." While pronouncing these last words, with great violence of gesture, and action of his arm, which he waved over his head, he struck his right hand with such force against a lamp which hung in the laboratory, that he roared with agony, and thrust his knuckles into his mouth to mitigate the *pain!*

*Physician.*—Ah! Friend Magendie! What say you to the evidence of the senses now? Here M. Magendie flounced out of the laboratory, and slammed the door in the physician's face, who was following him in a convulsion of laughter.

---

### Dr. Granville and Dr. Roe.

In our notice of Dr. Roe's work on Hooping-cough, in our last number, we made a mistake of twenty instead of ten years as the period of Dr. R's. experience of prussic acid, thus leaving Dr. Granville ten years of priority in the use of the remedy.

---

### Dr. Scholefield and Dr. Hooper.

In our Journal for October 1837, we took a short, but favourable notice of a little work on the Climate and Diseases of Jersey, by Dr. Hooper. It appears that Dr.

Scholefield furnished the Medical Chapter of Mr. Inglis's Work on the Channel-Islands, published in 1834, and he now comes forward to accuse Dr. Hooper of plagiarism, and of culling materials from his Chapter above-mentioned, without acknowledgment for his recent pamphlet. In the JERSEY TIMES, of the 23rd of November last, Dr. Scholefield has collated, in juxta-position, the original passages and those which he deems to be pirated by Dr. Hooper. We have not space for this controversy about priority; but, on glancing over this collation (not a cold one certainly) we apprehend that Dr. Scholefield has some reason to complain on this occasion—not of us—for our commendation of the pirate (if a pirate) was surely a compliment paid to the original writer.

---

### SCOTT ON COVE.

*To the Editors of the Medico-Chirurgical Review.*

GENTLEMEN.—May I beg to call your attention to one or two errors which crept into the notice of my paper upon the Medical Topography of Cove, and which you did me the favor to call the attention of the profession to in the Number just published of your valuable Journal. Your reviewer writes thus—" this is Spike Island at the entrance of Cove Harbour, &c." Now the sentence should be, *this is Cove in the County of Cork.* Spike Island is barely incidentally mentioned in my paper, and how the accident of substituting it for Cove could have occurred I do not know. Again, the Reviewer says at line 40, " the island and its village are sheltered from the winds, &c." This should be *the town* is sheltered from the winds, &c.; for it is the town of Cove that enjoys protection from the colder winds, and not the island. It is beside a large town, and it will give an idea of its size when I name its population at near 9000 souls. These errors I need only point your attention to, I am certain, to have corrected; as to your readers they would convey indeed a very erroneous idea of, without any partiality, one of the most beautifully and favorably situated towns that can be seen.

Your most obedient and obliged servant,

*Cove, July. 28th,* 1838. D. W. SCOTT.

---

### FURTHER TESTIMONY OF THE REMEDIAL VALUE OF THE CARBONATE OF SODA.

I have long prescribed it,—frequently in large doses; and without enumerating its beneficial action in typhus fever, in dyspepsia, certain states of anemia, &c., I will content myself with pointing out its great value in the treatment of phthisis. Some of the numerous cases in which I have prescribed it are on record in my recently published work on consumption and scrofula; and, so far from its having caused liquefaction of the blood, infiltration of the lungs, or pneumonia, I think I have great reason to laud its tonic effects in removing crude tubercles, as well as in obviating that state of superabundant carbon in the blood, and that sluggish condition of it, which I believe to obtain in phthisis, in some of its stages, and the existence of which I have, in that work, endeavoured to show. It has been especially useful in threatened cases of tubercular infiltration. It has not even appeared to increase the frequency of that intercurrent pneumonia which is so often observed to occur in phthisis still less to give rise to it; indeed, my experience would incline me to support the very reverse propositions.

The direct injection of the carbonate of soda into the blood is one thing; but the introducing it into the stomach is another; and whether or not it there meets with free hydrochloric acid, or is otherwise changed, the ultimate effect, as regards the blood, must be very different from that of direct injection; at all events, similarity or identity of effect must not be hastily assumed.

*Hertford, Nov.* 18, 1838. J. J. FURNIVALL, M.D.

